# Loss of Transient Receptor Potential Melastatin 3 ion channel function in natural killer cells from Chronic Fatigue Syndrome/Myalgic Encephalomyelitis patients

**DOI:** 10.1186/s10020-018-0046-1

**Published:** 2018-08-14

**Authors:** Hélène Cabanas, Katsuhiko Muraki, Natalie Eaton, Cassandra Balinas, Donald Staines, Sonya Marshall-Gradisnik

**Affiliations:** 1 School of Medical Science, Griffith University, Gold Coast, QLD Australia; 2 The National Centre for Neuroimmunology and Emerging Diseases, Menzies Health Institute Queensland, Griffith University, Gold Coast, QLD Australia; 3 Laboratory of Cellular Pharmacology, School of Pharmacy, Aichi-Gakuin University, Chikusa, Nagoya, Japan

**Keywords:** Transient receptor potential Melastatin 3, Calcium, Chronic fatigue syndrome/Myalgic encephalomyelitis, Natural killer cells, Flow cytometry, Patch-clamp

## Abstract

**Background:**

Chronic Fatigue Syndrome (CFS)/ Myalgic Encephalomyelitis (ME) is a debilitating disorder that is accompanied by reduced cytotoxic activity in natural killer (NK) cells. NK cells are an essential innate immune cell, responsible for recognising and inducing apoptosis of tumour and virus infected cells. Calcium is an essential component in mediating this cellular function. Transient Receptor Potential Melastatin 3 (TRPM3) cation channels have an important regulatory role in mediating calcium influx to help maintain cellular homeostasis. Several single nucleotide polymorphisms have been reported in *TRPM3* genes from isolated peripheral blood mononuclear cells, NK and B cells in patients with CFS/ME and have been proposed to correlate with illness presentation. Moreover, a significant reduction in both TRPM3 surface expression and intracellular calcium mobilisation in NK cells has been found in CFS/ME patients compared with healthy controls. Despite the functional importance of TRPM3, little is known about the ion channel function in NK cells and the epiphenomenon of CFS/ME. The objective of the present study was to characterise the TRPM3 ion channel function in NK cells from CFS/ME patients in comparison with healthy controls using whole cell patch-clamp techniques.

**Methods:**

NK cells were isolated from 12 age- and sex-matched healthy controls and CFS patients. Whole cell electrophysiology recording has been used to assess TRPM3 ion channel activity after modulation with pregnenolone sulfate and ononetin.

**Results:**

We report a significant reduction in amplitude of TRPM3 current after pregnenolone sulfate stimulation in isolated NK cells from CFS/ME patients compared with healthy controls. In addition, we found pregnenolone sulfate-evoked ionic currents through TRPM3 channels were significantly modulated by ononetin in isolated NK cells from healthy controls compared with CFS/ME patients.

**Conclusions:**

TRPM3 activity is impaired in CFS/ME patients suggesting changes in intracellular Ca^2+^ concentration, which may impact NK cellular functions. This investigation further helps to understand the intracellular-mediated roles in NK cells and confirm the potential role of TRPM3 ion channels in the aetiology and pathomechanism of CFS/ME.

## Background

Chronic Fatigue Syndrome/ Myalgic Encephalomyelitis (CFS/ME) is a complex and debilitating disorder hallmarked by persistent or relapsing chronic fatigue that is inadequately alleviated by rest (Fukuda et al., 1994). At least four concurrent symptoms related to multiple systems including the immune, neurological, musculoskeletal, gastrointestinal, and cardiovascular systems accompany this unexplained fatigue (Carruthers et al., 2011). Without a pathology test, diagnosis is complex and relies on different case definitions that address the characteristics of CFS/ME (Carruthers et al., 2011; Jason et al., 2015). Although the Centers for Disease Control and Prevention (CDC) criteria are widely employed, this definition has been considered too broad in its symptom requirements (Johnston et al., 2013). Thus, the definition of CFS/ME was revised producing the Canadian Consensus Criteria (CCC) and finally in 2011 a more specific definition was established known as the International Consensus Criteria (ICC) to assist diagnosis (Carruthers et al., 2011). The underlying aetiology of CFS/ME remains unknown. However, a consistent feature of CFS/ME in the literature is immune dysfunction, and more precisely a significant reduction in Natural Killer (NK) cell cytotoxicity, a hallmark of NK cell function (Brenu et al., 2012; Brenu et al., 2011; Curriu et al., 2013; Hardcastle et al., 2015; Huth et al., 2016a; Klimas et al., 1990; Maher et al., 2005; Natelson et al., 2002; Nijs & Frémont, 2008; Sharpe et al., 1991; Siegel et al., 2006; Stanietsky & Mandelboim, 2010).

NK cells are effector lymphocytes of the innate immune system that eliminate pathogens and tumour cells, in addition to immune cell activation and cytokine production (Vivier et al., 2008). NK cells can be divided into five different phenotypes determined by their expression of cell-surface molecules including CD56 and CD16. The two main mature NK cell subtypes are CD56^bright^CD16^dim^ and CD56^dim^CD16^bright^. CD56^bright^CD16^dim^ NK cells exist as the minority in peripheral blood as efficient cytokine producers (Cooper et al., 2001). The CD56^dim^CD16^bright^ subset constitutes 90% of human peripheral NK cells with significantly higher cytotoxic activity than CD56^bright^ NK cells as they contain an abundance of cytolytic proteins. Additionally, the presence of the low-affinity Fc-γ receptor CD16 facilitates the activation of antibody-dependent cellular cytotoxicity (ADCC) (Moretta, 2010; Stabile et al., 2015). Differences in NK cell phenotypes and significantly reduced peripheral NK cell numbers resulting in significant reduction in NK cell cytotoxicity, have been reported in CFS/ME and implicated in disease severity (Brenu et al., 2013; Hardcastle et al., 2015; Lanier, 2003; Maher et al., 2005; Nijs & Frémont, 2008). Importantly, NK cells require calcium (Ca^2+^) to regulate cellular functions including NK cell cytotoxicity. Indeed, numerous steps including adhesion to the target cell, activation of surface receptors, microtubule reorganisation, polarisation of secretory granules and release of lytic proteins, including granzyme A and granzyme B, creation of the immune synapse, formation of perforin pores, and finally granzyme-induced target cell apoptosis require tight regulation of Ca^2+^ signalling (Anasetti et al., 1987; Henkart, 1985; Kass & Orrenius, 1999; Schwarz et al., 2013).

Transient Receptor Potential (TRP) ion channels can trigger specific Ca^2+^-dependent signal transduction pathways regulating many biological processes in both excitable and nonexcitable cells (Gees et al., 2010). TRP channels represent a large and diverse family of nonselective cation channels that are widely expressed and respond to a wide range of chemical and physical stimuli (Voets et al., 2005). Moreover, genetic variations in TRP genes and noxious stimuli have been implicated in several pain-related pathological conditions/modalities, including inflammatory, neuropathic, visceral and dental pain, as well as pain associated with cancer (Mickle et al., 2016; Nilius, 2007). The mammalian TRP channels are divided into subgroups according to amino acid sequence similarities: TRPC (canonical), TRPM (melastatin), TRPV (vanilloid), TRPA (ankyrin), TRPML (mucolipin), and TRPP (polycystin) (Clapham et al., 2001). TRP cation channel subfamily M member 3 (TRPM3) is a Ca^2+^-permeable nonselective cation channel, expressing a calmodulin binding region on the N-terminus which plays a role in activation of Ca^2+^ dependent signalling pathways (Holakovska et al., 2012; Lee et al., 2003; Oberwinkler & Philipp, 2014). The human *TRPM3* gene encodes for many different TRPM3 isoforms due to alternative splicing and exon usage, leading to channels with divergent pore and gating properties (Frühwald et al., 2012; Oberwinkler et al., 2005; Thiel et al., 2013). In particular, the TRPM3α2 isoform has been characterized as being highly permeable for Ca^2+^ and other divalent cations (Frühwald et al., 2012). TRPM3 ion channels are highly expressed in neurons of dorsal root ganglia, where they serve as thermosensitive channels implicated in the detection of noxious heat (Vriens et al., 2011). Furthermore, TRPM3 has been identified in a number of tissues and cell types, including pancreatic beta cells, brain, pituitary gland, eye, kidney, and adipose tissue, that serve many different functions (Hoffmann et al., 2010; Oberwinkler & Philipp, 2014; Wagner et al., 2008). While expressed ubiquitously in mammalian cells, the roles and functions of TRPM3 have yet to be determined in immune cells and more particularly in NK cells, where TRPM3 has been previously identified without electrophysiological evaluation (Nguyen et al., 2017; Nguyen et al., 2016).

TRPM3 ion channels are quickly (< 100 ms) and reversibly activated by a neuronal steroid, Pregnenolone sulfate (PregS) (Wagner et al., 2008). The precursor, pregnenolone is derived from cholesterol and sulphated in vivo for biological actions in the immune and central nervous systems. It is associated with memory and cognition, neuronal myelination, activation of neurotransmitter-gated channels, modulation of glutamate–nitric oxide–guanosine 3′,5′-(cyclic) phosphate pathways, maintenance of glucose and insulin homeostasis and the management of noxious stimuli (Harteneck, 2013; Nilius & Voets, 2008). Stimulation of TRPM3 with PregS in pancreatic beta-cells induces an intracellular signalling cascade, involving a rise in intracellular Ca^2+^ concentration ([Ca^2+^]_i_), activation of the protein kinases Raf and extracellular signal-regulated kinases (ERK), resulting in the regulation of different cellular processes and a change in gene expression pattern (Thiel et al., 2013). However, it is notable that concentrations of PregS were sometimes high (30–100 μM), causing non-specific effects without TRPM3. On the other hand, a natural compound, deoxybezoin ononetin, has been identified as a selective and potent blocker of PregS-induced TRPM3 currents in TRPM3-expressing dorsal root ganglia neurones and TRPM3 transfected HEK293 cells (Straub et al., 2013). Therefore, both use of TRPM3 agonist and blocker are critical to identify the activity of TRPM3 currents in native cells.

Regulation and importance of TRPM3 channels in NK cells and the epiphenomenon of CFS/ME is relatively unknown. Five single nucleotide polymorphisms (SNPs) (rs6560200, rs1106948, rs12350232, rs11142822, rs1891301) have been identified in *TRPM3* genes in CFS/ME patients (Marshall-Gradisnik et al., 2016). A recent investigation characterising TRPM3 related responses in NK cells and B lymphocytes found a significant reduction in expression of TRPM3 on the NK cell surface in CFS/ME patients compared with healthy controls (HC) (Nguyen et al., 2016). Moreover, isolated NK cells from CFS/ME patients have impaired TRPM3 activity following PregS stimulation, resulting in impaired Ca^2+^ mobilisation and reduced NK cell cytotoxicity (Nguyen et al., 2017). These results strongly suggest the importance of TRPM3 in the pathophysiology of CFS/ME. However, as the electrophysiological characterisation of endogenous TRPM3 channels on isolated NK cells is lacking, we aimed to characterise TRPM3 channel currents using whole cell patch-clamp measurements in HC and CFS/ME patients after modulation with PregS and ononetin. This novel approach may help to understand the clinical importance of TRPM3 in the pathomechanism of CFS/ME.

## Methods

### Participant recruitment

Six CFS/ME patients and six age- and sex-matched HC were recruited using the National Centre for Neuroimmunology and Emerging Diseases (NCNED) research database. Participants were screened using a comprehensive questionnaire corresponding with the CDC, CCC and ICC case definitions. All six CFS/ME patients were defined by the CCC. HC reported no incidence of fatigue and were in good health without evidence of illness. Participants were excluded from this study if they reported history of smoking, autoimmune diseases, cardiac disease, diabetes or other co-morbidities.

Two of the six CFS/ME patients reported regular administration of non-steroidal anti-inflammatory for pain relief. No participants reported use of pharmacological agents that directly or indirectly influence TRPM3 or Ca^2+^ signalling.

This investigation was approved by the Griffith University Human Research Ethics Committee (HREC/15/QGC/63).

### Peripheral blood mononuclear cell isolation and natural killer cell isolation

A total of 85 ml of whole blood was collected in ethylendiaminetetraacetic acid (EDTA) tubes between 8:00 am and 12:00 am. Routine full blood analysis was performed within 4h of collection for red blood cell count, white blood cell count and granulocyte cell count.

Peripheral blood mononuclear cells (PBMCs) were isolated from 80 ml of whole blood by centrifugation over a density gradient medium (Ficoll-Paque Premium; GE Healthcare, Uppsala, Sweden) as previously described (Brenu et al., 2011; Munoz & Leff, 2006). PBMCs were stained with trypan blue (Invitrogen, Carlsband, CA, USA) to determine cell count and cell viability. PBMCs were adjusted to a final concentration of 5 × 10^7^ cells/ml for NK cell isolation.

NK cells were isolated by immunomagnetic selection using an EasySep Negative Human NK Cell Isolation Kit (Stem Cell Technologies, Vancouver, BC, Canada). NK Cell purification was determined using flow cytometry. NK cells were incubated for 20 min at room temperature in the presence of CD56 FITC (0.25 μg/5 μl) and CD3 PE Cy7 (0.25 μg/20 μl) monoclonal antibodies (BD Bioscience, San Jose, CA, USA) as previously described (Nguyen et al., 2017).

### Whole cell electrophysiology recording

Borosilicate glass capillaries with an outside diameter of 1.5 mm and inside diameter of 0.86 mm (Harvard Apparatus, Holliston, MA, USA) were used as patch pipettes. Pipette resistance when filled with pipette solution was 8–12 MΩ. The pipettes were mounted on a CV203BU head-stage (Molecular Devices, Sunnyvale, CA, USA) connected to a 3-way coarse manipulator and a micro-manipulator (Narishige, Tokyo, Japan). Electrical signals were amplified and recorded using an Axopatch 200B amplifier and PClamp 10.7 software (Molecular Devices, Sunnyvale, CA, USA). Data were filtered at 5 kHz and sampled digitally at 10 kHz via a Digidata 1440A analogue to digital converter (Molecular Devices, Sunnyvale, CA, USA). The voltage-ramp protocol was a step from a holding potential of + 10 mV to − 90 mV, followed by a 0.1 s ramp to + 110 mV, before returning to + 10 mV (repeated every 10 s). The liquid junction potential between the pipette and bath solutions (− 10 mV) was corrected. A leak current component was not subtracted from the recorded currents. Electrode was filled with the intracellular pipette solution containing 30 mM CsCl, 2 mM MgCl_2_, 110 mM L-Aspartic acid, 1 mM EGTA, 10 mM HEPES, 4 mM ATP, 0.1 mM GTP, adjusted pH to 7.2 with CsOH and osmolality of 290 mOsm/L with D-mannitol. The pipette solution was filtered using a 0.22 μm membrane filter (Sigma-Aldrich, St. Louise, MO, USA), divided into aliquots and stored at − 20 °C. Bath solution contained: 130 mM NaCl, 10 mM CsCl, 1 mM MgCl_2_, 1.5 mM CaCl_2_2H_2_O, 10 mM HEPES, adjusted pH to 7.4 with NaOH and osmolality 300 mOsm/L with D-glucose. All reagents were purchased from Sigma-Aldrich, except for ATP and GTP that were purchased from Sapphire Bioscience. TRPM3 currents were stimulated by adding 100 μM PregS (Tocris Bioscience, Bristol, UK) to the bath solution, whereas PregS-induced TRPM3 currents were blocked by adding 10 μM ononetin (Tocris Bioscience, Bristol, UK). All measurements were performed at room temperature. The authors removed the possibility of chloride current involvement in TRPM3 assessment by using L-Aspartic acid in the intracellular pipette solution. Unstable currents were also removed from the analysis. This ensured only TRPM3 function was assessed.

### Statistical analysis

**Fig. 1:**
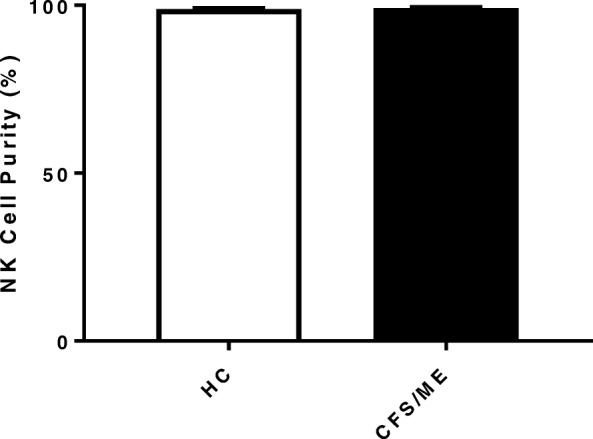
Natural Killer cell purity**.** Bar graphs representing isolated NK cell purity for HC and CFS/ME patients. Data presented as mean ± SEM. HC = 98.07% ± 0.80 and CFS/ME = 98.28% ± 0.58. Abbreviations: CFS/ME, chronic fatigue syndrome/myalgic encephalomyelitis; HC, healthy controls; NK cell, natural killer cell

Cytometry data was exported from FacsDiva v8.1 and analysed using SPSS v24 (IBM Corp, Version 24, Armonk, NY, USA) and GraphPad Prism v7 (GraphPad Software Inc., Version 7, La Jolla, CA, USA). Electrophysiological data were analysed using pCLAMP 10.7 software (Molecular Devices, Sunnyvale, CA, USA). Origin 2018 (OriginLab Corporation, Northampton, MA, USA) and GraphPad Prism v7 (GraphPad Software Inc., Version 7, La Jolla, CA, USA) were used for statistical analysis and data presentation. The Shapiro-Wilk test was used to determine population normality. Statistical comparison was performed using the Mann-Whitney U test (Table 1, Fig. 1 and Fig. 3e), and Fishers exact test (Fig. 3g), to determine any significant differences. Significance was set at *p* < 0.05 and the data are presented as mean ± SEM unless otherwise stated.

**Table 1:** Blood parameters and patient demographic

	CFS/ME	HC	*P* Value
Age (years)	42.5 ± 6.05	42.8 ± 5.47	0.872
Gender n(%)			
Male	2 (20%)	2 (20%)	1.000
Female	4 (80%)	4 (80%)	
BMI (kg/m^2^)	23.54 ± 0.54	23.35 ± 1.37	0.240
SF-36			
Fatigue (%)	30.42 ± 8.91	77.92 ± 6.25	*0.009*
General Health (%)	31.25 ± 5.97	74.31 ± 4.50	*0.004*
Physical Functioning (%)	70.83 ± 6.64	93.33 ± 4.77	*0.015*
Role Physical (%)	20.83 ± 9.64	87.50 ± 12.5	*0.009*
Role Emotional (%)	59.38 ± 9.24	96.88 ± 3.13	*0.004*
Social Functioning (%)	37.5 ± 5.59	95.83 ± 4.17	*0.002*
Body Pain (%)	49.17 ± 5.27	84.17 ± 8.41	*0.015*
Pathology			
White Cell Count (×10^9^/L)	5.58 ± 0.30	7.13 ± 0.49	*0.03*
Lymphocytes (×10^9^/L)	1.99 ± 0.2	1.75 ± 0.22	0.423
Neutrophils (× 10^9^/L)	3.03 ± 0.23	4.78 ± 0.42	*0.01*
Monocytes (× 10^9^/L)	0.41 ± 0.05	0.39 ± 0.06	0.748
Eosinophils (× 10^9^/L)	0.12 ± 0.02	0.18 ± 0.06	0.936
Basophils (× 10^9^/L)	0.04 ± 0.01	0.04 ± 0.002	0.799
Platelet (× 10^9^/L)	265.0 ± 23.36	266.17 ± 15.70	1.00
Red Cell Count (× 10^12^/L)	4.63 ± 0.09	4.69 ± 0.13	0.63
Haematocrit	0.41 ± 0.01	0.42 ± 0.01	0.624
Haemoglobin (g/L)	135.0 ± 3.52	139.67 ± 5.21	0.687

SF-36 scores were analysed using participant questionnaire responses. Results from routine full blood analysis in CFS/ME patients and HC. Data presented as mean ± SD. *Abbreviations: CFS/ME, chronic fatigue syndrome/myalgic encephalomyelitis; HC, healthy controls; BMI, body mass index*

## Results

### Participant characteristics and blood parameters

A total of twelve age- and sex-matched participants were included for this investigation. Demographic and clinical data for patients are summarised in Table 1. There was no significant difference in age or gender between patients and HC. The 36-Item Short Form Survey (SF-36) was used to determine participant health-related-quality of life. As expected, there was a significant difference in SF-36 scores between CFS/ME patients and HC. Moreover, while there was a significant difference in white cell count and neutrophils, all results remained within normal range for age and sex as provided by the Gold Coast University Hospital Pathology Unit, NATA accredited laboratory.

### Natural killer cell purity

NK cell purity (CD3^−^/CD56^+^) was 98.07% ± 0.80 for HC and 98.28% ± 0.58 for CFS/ME patients (Fig. 1) as determined by flow cytometry. There was no significant difference in NK cell purity in CFS/ME patients compared with HC.

### TRPM3 activity after Pregnenolone sulfate stimulation

**Fig. 2:**
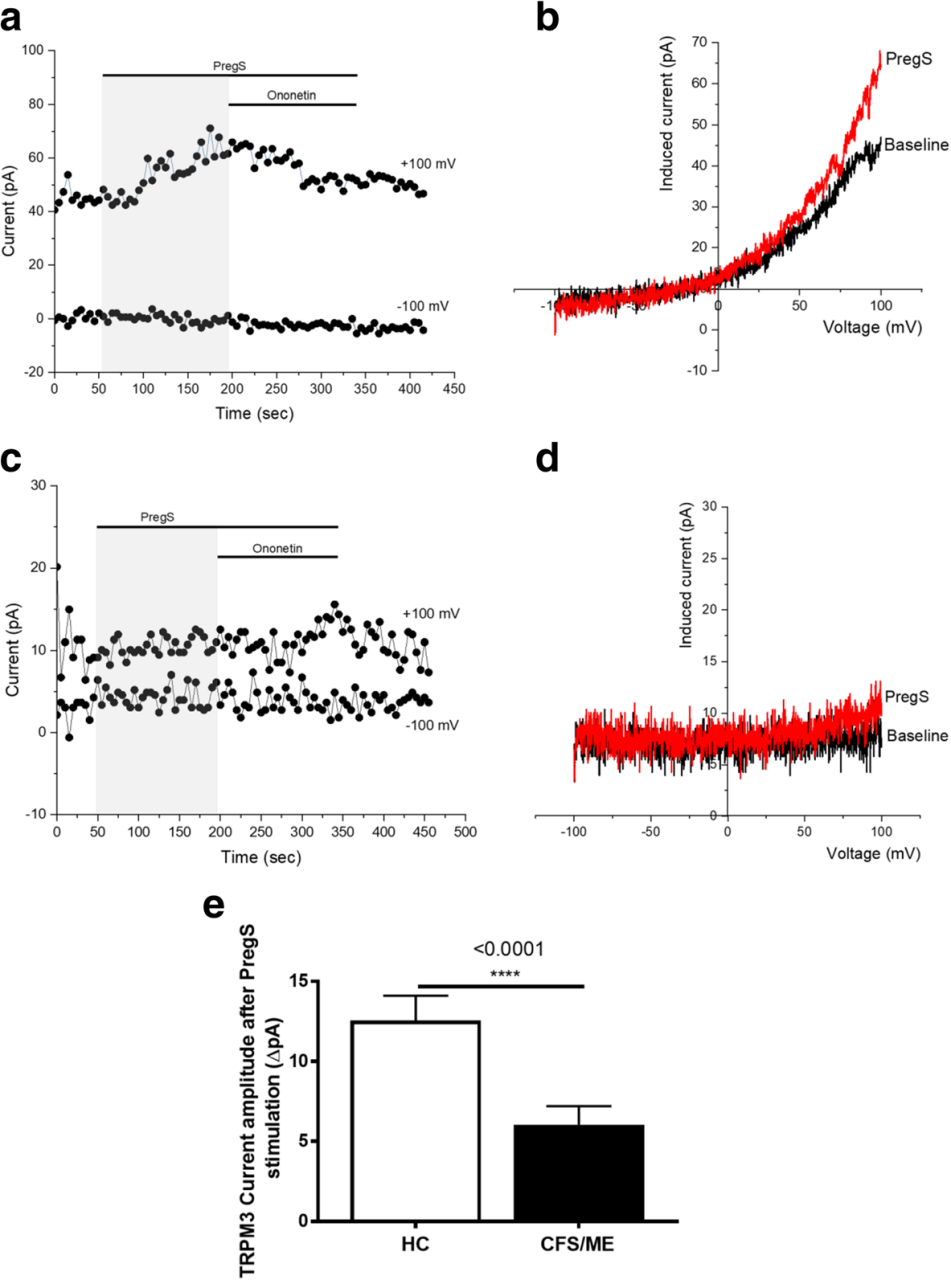
TRPM3 activity after PregS stimulation. Data were obtained under whole-cell patch clamp conditions. **a** A representative time-series of current amplitude at + 100 mV and − 100 mV showing the effect of 100 μΜ PregS on ionic currents in isolated NK cells from HC. **b** *I*–*V* before and after PregS stimulation in a cell corresponding with (**a**.)**. c** A representative time-series of current amplitude at + 100 mV and − 100 mV showing the effect of 100 μΜ PregS on ionic currents in isolated NK cells from CFS/ME patients. **d.** *I*–*V* before and after PregS stimulation in a cell as shown in (**c**.)**. e** Bar graphs representing TRPM3 current amplitude at + 100 mV after stimulation with 100 μΜ PregS in CFS/ME patients (*N* = 6; *n* = 33) compared with HC (N = 6; *n* = 29). Data are represented as mean ± SEM. Abbreviations: CFS/ME, chronic fatigue syndrome/myalgic encephalomyelitis; HC, healthy control; NK, natural killer

A whole-cell patch-clamp technique was used to measure endogenous TRPM3 activity, enabling a small size of current recordings under voltage-clamp conditions and observation of the typical shape of the TRPM3 current–voltage relationship (*I*–*V*) (Fig. 2). We used 100 μM PregS to stimulate the channels as TRPM3 is minimally expressed in NK cells (Nguyen et al., 2017; Nguyen et al., 2016). As expected, the ionic current evoked by PregS was relatively small (Fig. 2a and e) in NK cells isolated from HC and we report a typical *I*–*V* of TRPM3, which had a clear outward rectification (Fig. 2b). In contrast, the amplitude of ionic current after PregS stimulation was significantly smaller (Fig. 2c, d) in NK cells from CFS/ME patients than that from HC (Fig. 2e, *p* < 0.0001), suggesting impaired TRPM3 channel activity after PregS stimulation in CFS/ME patients.

### TRPM3 activity after ononetin modulation

**Fig. 3:**
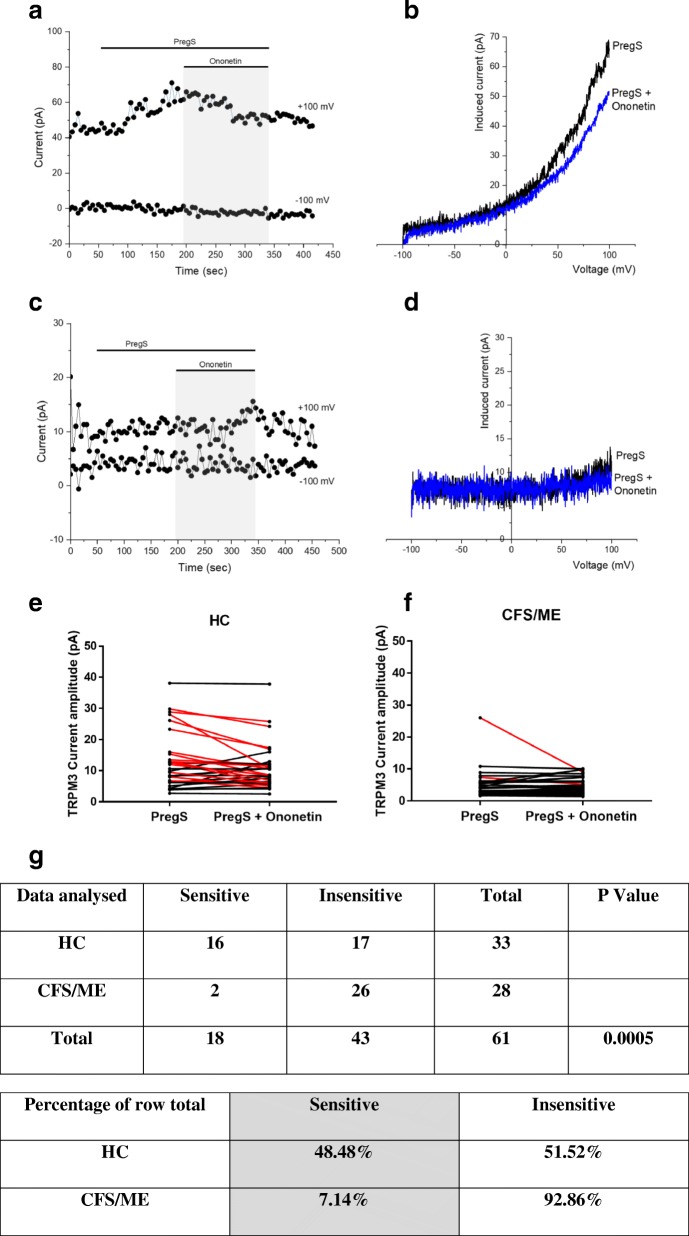
TRPM3 activity after ononetin modulation**.** Data were obtained under whole-cell patch clamp conditions. **a.** A representative time-series of current amplitude at + 100 mV and − 100 mV showing the effect of 10 μΜ ononetin on ionic currents in the presence of PregS in isolated NK cells from HC. **b** *I*–*V* before and after application of ononetin in a cell as shown in (**a**.)**. c** A representative time-series of current amplitude at + 100 mV and − 100 mV showing the effect of 10 μΜ ononetin on ionic currents in the presence of PregS in isolated NK cells CFS/ME patients. **d.** *I*–*V* before and after application of ononetin in a cell as shown in (**c**.)**. e. f** Scatter plots representing change of each current amplitude before and after ononetin application in all NK cells from HC and CFS/ME patients. Each cell represented as red lines had reduction in currents by ononetin. **g** Table summarizing data for sensitive and insensitive cells to 10 μΜ ononetin in HC (N = 6; n = 33) compared to CFS/ME patients (N = 6; *n* = 28). Data are analysed with Fisher’s exact test. Abbreviations: CFS/ME, chronic fatigue syndrome/myalgic encephalomyelitis; HC, healthy controls

Ononetin effectively inhibits PregS-evoked Ca^2+^-influx and ionic currents through TRPM3 channels (Straub et al., 2013). Therefore, to confirm that TRPM3 activity is involved in ionic currents evoked by PregS in NK cells, we next used 10 μM ononetin to modulate the channels (Fig. 3). As shown in Fig. 3a, the ionic currents evoked by PregS were effectively inhibited by simultaneous application of ononetin in isolated NK cells from HC. Moreover, the *I*–*V* of ononetin sensitive currents was outwardly-rectified and typical for TRPM3 (Fig. 3b). In contrast, ionic currents in the presence of PregS were mostly resistant to ononetin in isolated NK cells from CFS/ME patients (Fig. 3c, d), in comparison with HC (Fig. 3e, f, g (*p* = 0.0005)), showing significant loss of the TRPM3 channel activity in CFS/ME patients.

## Discussion

Our previous investigations have proposed that NK cells from CFS/ME patients have significantly reduced expression of TRPM3 and subsequent reduction in intracellular Ca^2+^ mobilisation compared with HC (Nguyen et al., 2017; Nguyen et al., 2016). This present study used electrophysiological methods to characterise endogenous TRPM3 activity in peripheral NK cells from HC and CFS/ME patients. We provide evidence suggesting PregS-dependent channel activity for TRPM3 is significantly lower in CFS/ME patients compared with HC. Moreover, ionic currents in CFS/ME patients were resistant to ononetin in the presence of PregS.

The patch clamp technique is regarded as a gold standard for ion channel research and offers direct insight into ion channel properties through the characterization of ion channel activity. In this study, we characterised, for the first time, the TRPM3 ion channel current in isolated human NK cells. We report a significant reduction amplitude of TRPM3 current after PregS stimulation in isolated NK cells from CFS/ME patients compared with HC. This is consistent with our previous findings showing significantly reduced TRPM3 expression as well as significantly reduced intracellular Ca^2+^ mobilisation in isolated NK cells from CFS/ME patients compared with HC (Nguyen et al., 2017; Nguyen et al., 2016). In addition, we found PregS-evoked ionic currents through TRPM3 channels were significantly modulated by ononetin in isolated NK cells from HC compared with CFS/ME patients. Indeed, isolated NK cells from CFS/ME were resistant to ononetin suggesting that PregS may activate non-TRPM3 cationic currents in CFS/ME patients. Alternatively, CFS/ME patients may express different spliced isoforms of TRPM3 that are non-sensitive to ononetin. Although we demonstrate TRPM3 channel activity dysfunction in CFS/ME patients, further investigations are required to elucidate the mechanisms involved in the impaired TRPM3 channel activity as well as the different TRPM3 isoform types that are expressed in NK cells.

Previous electrophysiological investigations have shown that TRPM3 forms an ion channel permeable to Ca^2+^, sodium (Na^+^), magnesium (Mg^2+^), and manganese (Mn^2+^) (Grimm et al., 2005; Oberwinkler et al., 2005). Ca^2+^ plays an important role in intracellular signalling pathways, cell differentiation and division, apoptosis, and transcriptional events. In non-excitable cells, such as immune cells, a main Ca^2+^ entry pathway is known as store-operated Ca^2+^ entry (SOCE) and some TRP ion channels are associated with this pathway. While the sub-family TRPC have been traditionally associated with this important cellular mechanism (Cheng et al., 2013; Ong et al., 2016; Salido et al., 2009), recent research has also identified TRPM3 as a component for SOCE in white matter of the central nervous system (CNS) (Papanikolaou et al., 2017). Upon TRPM3 channel activation, changes in [Ca^2+^]_i_ occur, resulting in the regulation of many biological processes that correspond to an array of cells expressing this channel. TRPM3 is located and linked to vascular smooth muscle contraction, modulation of glucose-induced insulin release from pancreatic islets, detection of noxious heat in dorsal root ganglia and development of epithelial cells of the choroid plexus, as well as function of oligodendrocytes and neurons (Hoffmann et al., 2010; Oberwinkler et al., 2005; Vriens et al., 2011; Wagner et al., 2008). Therefore, dysregulation of TRPM3 family, affecting SOCE and more generally, Ca^2+^ signalling has significant implications for cell regulatory machinery.

Significant reduction in NK cell cytotoxicity is a consistent feature reported in CFS/ME patients (Brenu et al., 2012; Brenu et al., 2011; Curriu et al., 2013; Hardcastle et al., 2015; Huth et al., 2016a; Klimas et al., 1990; Maher et al., 2005; Natelson et al., 2002; Nijs & Frémont, 2008; Sharpe et al., 1991; Siegel et al., 2006; Stanietsky & Mandelboim, 2010). NK cell cytotoxic activity is a Ca^2+^ dependent process, which drives the intracellular microtubule reorganisation, polarisation of cytoplasmic granules, release of lytic proteins and the creation of the immune synapse (Anasetti et al., 1987; Henkart, 1985). Moreover, Ca^2+^-dependent cytotoxic processes allow for the production and recruitment of lytic proteins (Voskoboinik et al., 2015). Following cytotoxic granule delivery to the immune synapse, the formation of perforin pores and granzyme-induced cell apoptosis are highly dependent on Ca^2+^ (Orrenius et al., 2003; Voskoboinik et al., 2005). Previous studies have reported impaired Ca^2+^ signalling in NK cells from CFS/ME patients demonstrated through changes to ERK1/2 and mitogen-activated protein kinase (MAPK) pathways (Chacko et al., 2016; Huth et al., 2016b). CFS/ME patients have significantly decreased ERK1/2 following incubation with K562 cells (Huth et al., 2016b). ERK1/2 is activated in a Phosphatidylinositol-4,5-bisphosphate 3-kinase (P1_3_K)-dependent manner that may also be associated with cytoplasmic Ca^2+^ ion levels through activation of TRPM3 (Lee et al., 2003). In the absence of phosphatidylinositol 4,5-biphosphate (PIP_2_), TRPM3 is not activated, resulting in reduced cytosolic Ca^2+^ (Tóth et al., 2015). Previous research completed by Nguyen and colleagues reported that the expression of TRPM3 ion channel and [Ca^2+^]_i_ were significantly reduced in NK cells from CFS/ME patients (Nguyen et al., 2017). In addition, ERK1/2 requires Ca^2+^ as the final activator to initiate NK cell lysis (Huth et al., 2016b). Changes in Ca^2+^ signalling may impair cytokine production, including Interferon (IFN)-γ and Tumor Necrosis Factor (TNF), therefore interfering with systemic inflammation and anti-tumour responses (Romee et al., 2013). Previous investigations have reported both an increased and decreased inflammatory profile of CFS/ME patients along with reduced IFN-γ (Klimas et al., 1990; Lorusso et al., 2009). TRPM3-related Ca^2+^ dysfunction may then result in a reduction of [Ca^2+^]_i_, which may lower the function and cytotoxic capacity of the NK cells in CFS/ME patients.

Importantly, TRPM3 ion channels have a role in the detection of heat and in pain transmission in the CNS (Held et al., 2015). TRPM3 has been previously identified as a nociceptor channel involved in acute heat sensing and inflammatory heat hyperalgesia, and thus as a potential target for analgesic treatments (Vriens et al., 2011). Dysregulation of thermoregulatory responses has been reported in CFS/ME patients (Wyller et al., 2007). Generalised pain is a characteristic of CFS/ME and occurs in the absence of overt tissue damage, and this is suggestive of potential CNS impairments (Barnden et al., 2015; Barnden et al., 2016; Shan et al., 2016; Shan et al., 2017). Our present findings suggest TRPM3 ion channels may be involved in the pathomechansim of CFS/ME and hence have a possible role in nociception and thermoregulation.

While this study provides evidence of TRPM3 channel activity dysfunction in CFS/ME patients, this study is not without limitations with the low sample numbers. Indeed, these findings need to be validated in a larger cohort of patients. Additionally, the use of high and single doses of PregS and Ononetin may reduce the drugs specificity and selectivity and consequently modulate other non-TRPM3 cationic currents. Further investigations are also required to assess the impact of PregS and Ononetin on the different TRPM3 isoforms. This could explain the differences observed between HC and CFS/ME patients as well as within groups. Finally, TRPM4, TRPM5, TRPM2 and TRPM7 surface expression has been reported on B cells, bone marrow cells, splenic cells, lymph node B cells and T and mast cells (Zierler et al., 2017), suggesting our recent findings may also be pertinent to other TRPM channel functions and Ca^2+^ − mediated roles, such as SOCE.

## Conclusions

We have demonstrated impaired TRPM3 activity in CFS/ME patients through electrophysiological investigations in NK cells after modulation with PregS and ononetin. As TRPM3 is widely distributed in the body, particularly brain, eye, cardiovascular system, gastrointestinal system and pancreas, we assert that widespread changes in TRPM3 function in key body systems may contribute to CFS/ME. We suggest changes to Ca^2+^ ion concentration in the cytosol and intracellular stores result from changes in TRPM3 function, which may impact NK cellular functions. Therefore, Ca^2+^ signalling pathways could be an alternative therapeutic target in CFS/ME because of their importance in various cellular processes. This investigation confirms the potential role of TRPM3 ion channels in the aetiology and pathomechanism of CFS/ME, and could suggest potential therapeutic targets and/or prognostic markers.

## Abbreviations

[Ca^2+^]_i_: Intracellular Ca^2+^ concentration
ADCC: Antibody-dependent cellular cytotoxicity
BMI: Body Mass Index
Ca^2+^: Calcium
CCC: Canadian Consensus Criteria
CDC: Centers for Disease Control and Prevention
CFS/ME: Chronic Fatigue Syndrome/Myalgic Encephalomyelitis
CNS: Central nervous system
EDTA: Ethylendiaminetetraacetic acid
ERK: Extracellular signal-regulated kinases
HC: Healthy controls
ICC: International Consensus Criteria
IFN: Interferon
MAPK: Mitogen-activated protein kinase
Mg^2+^: Magnesium
Mn^2+^: Manganese
Na^+^: Sodium
NCNED: National Centre for Neuroimmunology and Emerging Diseases
NK: Natural killer
PBMCs: Peripheral blood mononuclear cells
PI_3_K: Phosphatidylinositol-4, 5-bisphosphate 3-kinase
PIP_2_: Phosphatidylinositol 4,5-bisphosphate
PregS: Pregnenolone sulfate
SF-36: 36-Item Short Form Survey
SNPs: Single nucleotide polymorphisms
SOCE: Store-operated Ca^2+^ entry
TNF: Tumour necrosis factor
TRP: Transient Receptor Potential
TRPA: Transient Receptor Potential Ankyrin
TRPC: Transient Receptor Potential Canonical
TRPM: Transient Receptor Potential Melastatin
TRPML: Transient Receptor Potential Mucolipin
TRPP: Transient Receptor Potential POlycystin
TRPV: Transient Receptor Potential Vanilloid
